# Sex differences in antibody responses to influenza A/H3N2 across the life course

**DOI:** 10.21203/rs.3.rs-7960157/v1

**Published:** 2025-11-17

**Authors:** Siyu Chen, Bernardo García-Carreras, James A. Hay, Huachen Zhu, Chao Qiang Jiang, Kin On Kwok, Steven Riley, Jonathan Read, Justin Lessler, Derek A.T. Cummings, C. Jessica E. Metcalf

**Affiliations:** 1 High Meadows Environmental Institute, Princeton University, Princeton, NJ, USA; 2 Department of Public and Ecosystem Health, College of Veterinary Medicine, Cornell University, Ithaca, NY, USA; 3 Department of Biology, University of Florida, Gainesville, FL 32611, USA; 4 Emerging Pathogens Institute, University of Florida, Gainesville, FL, USA; 5 Pandemic Sciences Institute, Nuffield Department of Medicine, University of Oxford, Oxford, UK; 6 School of Public Health, Li Ka Shing Faculty of Medicine, The University of Hong Kong, 21 Sassoon Road, Pokfulam, Hong Kong Special Administrative Region, China; 7 Guangzhou No. 13 Hospital, Guangzhou, Guangdong, 510620, China; 8 The Jockey Club School of Public Health and Primary Care, The Chinese University of Hong Kong, Hong Kong Special Administrative Region, China; 9 Hong Kong Institute of Asia-Pacific Studies, The Chinese University of Hong Kong, Hong Kong Special Administrative Region, China; 10 United Kingdom Health Security Agency, London, E14 4PU, United Kingdom; 11 MRC Centre for Global Infectious Disease Analysis, Jameel Institute for Disease and Emergency Analytics, Imperial College London, London, UK; 12 Lancaster Medical School, Lancaster University, Lancaster, LA1 4YW, United Kingdom; 13 Carolina Population Center, University of North Carolina at Chapel Hill, Chapel Hill, NC, USA; 14 Department of Epidemiology, Gillings School of Global Public Health, University of North Carolina at Chapel Hill, Chapel Hill, NC, USA; 15 Department of Epidemiology, Johns Hopkins Bloomberg School of Public Health, Baltimore, MD, USA; 16 Department of Epidemiology, Johns Hopkins Bloomberg School of Public Health, Baltimore, MD, USA; 17 Department of Biomedical Engineering, Johns Hopkins Whiting School of Engineering, Baltimore, MD, USA; 18 Department of Ecology and Evolutionary Biology, Princeton University, Princeton, NJ, USA; 19 School of Public and International Affairs, Princeton University, Princeton, NJ, USA

**Keywords:** influenza A/H3N2, hemagglutination inhibition antibody, sex, age, cross-reactivity

## Abstract

Sex differences in both innate and adaptive immunity are increasingly recognized as shaping responses to a range of pathogens. For antigenically variable pathogens that infect people many times over their lifetime, like influenza, sex differences may accumulate or be obscured by the interplay of immunity derived from each infection and future risk. However, sex-specific lifetime trajectories of influenza immunity are poorly characterized. Here, we analyzed hemagglutination inhibition antibody titers to multiple strains of influenza A/H3N2, measured in individuals spanning a wide age range to disentangle strain-specific and cross-reactive antibody responses by sex. To account for potential differences in exposure, we separately analyzed antibody titers to: (1) viruses circulating during an individual’s lifetime, and (2) viruses isolated before birth (pre-birth) or after sampling, with responses to the latter interpreted as exclusively cross-reactive, given the absence of possible exposure to these strains. We found that females aged 15 to 40 generally have higher antibody titers than males against pre-birth and post-sampling strains, consistent with greater cross-reactivity. Conversely, older males have stronger responses to strains that they could have encountered during their lives, suggesting that they mount stronger responses upon infection.

## Introduction

Sex differences in infectious disease outcomes are an increasing focus of public health research and practice [1–3]. Chromosomal and genetic regulation [4] and hormonal effects [5] modulate both innate and adaptive immunity. Adaptive immunity, in particular, may exert a strong influence on health over the life course [6]. As existing immune memory modulates both subsequent risk of infection and adaptive immune responses upon infection or vaccination, differences may be amplified leading to greater differences between sexes, particularly in pathogens undergoing evolution. However, some differences may be obscured over a life course, as gaps or weaknesses in an individual’s adaptive response may preferentially lead to infections and immune responses that fill these gaps. Here, we present an assessment of sex differences in antibody responses to multiple influenza A strains among individuals of varying ages to determine if persistent differences are seen and to assess whether these differences vary over the life course, reflecting the effect of amplification or mitigation of differences over multiple exposures.

Much of what is known about sex-differentiated influenza antibody responses comes from vaccine studies which often include repeated measurements following multiple vaccinations in both humans and animal models [7–8]. For example, in humans, receipt of seasonal influenza vaccine in adults 18–49 years of age results in hemagglutination inhibition (HI) antibody titers twice as high in females than in males [9]. In mice, comparison of cross-protection against an H1N1 drift variant following H1N1 vaccination found greater cross-protection in females, with strong evidence that antibodies alone mediated these effects [10].

While the immune consequences of vaccination may provide some insight into the immune consequences of natural infection, there are important factors that might alter age and sex trajectories of antibody responses: from differences in vaccination antigen dose relative to infection dose, to differences in site of exposure, to the trajectory of infection itself. Evidence on sex differences in responses and consequences of natural infection across the life course remains relatively limited. A small opportunistic study of natural infection with the 2009 H1N1 pandemic strain involving people aged 60 years or older in Australia found that the prevalence of cross-reactive antibodies was highest in the oldest age groups (≥ 85 years), especially for females aged 80–84 years, where 50% of women had an HI antibody titer ≥ 1:40 compared with only 17% of men [11]. Such differences might also translate into differences in susceptibility to infection: for example, another Australian study suggested that females in some age groups may be more susceptible to certain subtypes of influenza virus, even after accounting for health seeking behaviors [12]. Both studies suggest that the interaction between age and sex plays an important role in shaping immune responses against seasonal influenza.

Influenza undergoes repeated, punctuated antigenic evolution, resulting in immune escape and thus affecting the composition of an individual’s infection history. In response to selection by adaptive immunity, the surface glycoproteins of influenza viruses experience ‘antigenic drift’, moving between ‘antigenic clusters’ [13]. Subsequent strains tend to outcompete previous strains given their greater capacity to evade adaptive immunity, so that there is limited diversity of viruses at any time, and different A/H3N2 strains circulate at mutually exclusive times [14]. An individual’s immune memory to influenza is driven by prior exposures through a collection of phenomena including immune imprinting [15–16] and antigenic seniority [17–18]. These immunological responses together with patterns of immune ontogeny and senescence may all differ by sex [19]. Differentiation of strain specific and cross-reactive antibody responses between the sexes provides a window onto these processes. Here, we use a wide age-range, cross-sectional and longitudinal survey of HI titers to an antigenically broad panel of influenza A/H3N2 strains among people living in Guangzhou, China to understand sex differences in antibody responses to influenza A/H3N2 across the life course. We compare and contrast strains that individuals had never had exposure to (either because they circulated before the individual was born, or after the sample was taken) and with strains that circulated during their lifetime.

## Materials and methods

### Ethical approval

The following institutional review boards approved the study protocols: Johns Hopkins Bloomberg School of Public Health (IRB 1716), University of Florida (IRB201601953), University of Liverpool, University of Hong Kong (UW 09–020), and Guangzhou No. 12 Hospital (‘Research on human influenza virus immunity in Southern China’). Written informed consent was obtained from all participants over 12 years old; verbal assent was obtained from participants 12 years old or younger. Written permission from a legally authorized representative was obtained for all participants under 18 years old.

### Cohort and serological data

We used serum sampled starting in 2009, from 1130 participants who were recruited to the ongoing Fluscape cohort, a large-scale, cross-sectional and longitudinal serological study in Guangzhou, China. All participants provided a first blood sample from 2009 to 2012 while 1108 provided a second blood sample from 2013 to 2015 [20]. The cohort was recruited at 40 locations randomly distributed across a sector shaped area spanning from the center of the city of Guangzhou to neighboring rural areas. Antibody titers against 21 A(H3N2) strains using HI assays were measured. Strains tested were isolated from 1968 to 2014 including A/Hong Kong/1968, X-31 (isolated in 1970), A/England/1972, A/Victoria/1975, A/Texas/1977, A/Bangkok/1979, A/Philippines/1982, A/Mississippi/1985,A/Sichuan/1987, A/Beijing/1989, A/Beijing/1992, A/Wuhan/1995,A/Victoria/1998, A/Fujian/2000, A/Fujian/2002, A/California/2004, A/Brisbane/2007, A/Perth/2009, A/Victoria/2009, A/Texas/2012, and A/Hong Kong/2014 [21]. Vaccination uptake in this cohort was low with only 15 (1.3%) participants self-reporting influenza vaccination at any point during follow up. Detailed laboratory methods have been described previously [6,21].

### Antibody diversity metrics

We first examined how HI responses to the ensemble of 21 strains varied as function of age and sex among participants. Yang et al (2020) [6] compiled a number of summary metrics applied to HI responses of a subset of participants that we consider here, in particular ‘width’, representing the span of strains in antigenic space for which an individual had protective (≥1:40) or detectable titers (≥1:10) and ‘area under the curve (AUC)’, representing an individual’s total antibody response to all tested strains (see Supplementary Information).

### Identifying cross-reactivity

Antibody titers recorded for each viral strain in a participant’s sample is the outcome of two processes: 1) strain-specific antibody responses, reflecting that individual’s response to the tested strain originating from exposure to that strain; 2) cross-reactive antibody responses generated by exposure to antigenically similar strains. Thus, a purely cross-reactive antibody response will be reflected in the magnitude of antibody titers against influenza strains to which an individual could never have been exposed, which might be i) strains that circulated before the participant was born (‘backward cross-reactivity’); or ii) strains that were isolated after the participant provided a serum sample (‘forward cross-reactivity’).

**Figure 1.**
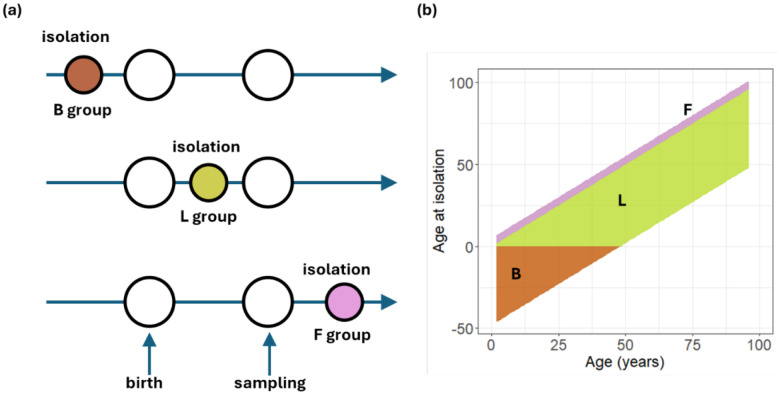
Schematic of different participant/virus groups used for analysis illustrating relative timing of strain isolation, participant birth, and sampling. a) the B group corresponds to viral isolation occurring prior to Birth, so that there is negative age-at-isolation; In the L group, viral isolation occurred after participant birth (during Lifetime) but prior to sampling, i.e., both time-since-isolation and age-at-isolation are positive. In the F group, viral isolation occurred after sampling (Future viruses) resulting in a positive age-at-isolation and a negative time-since-isolation. (b) Composition of tested samples in the color-coded three groups by participants’ age at sampling (x-axis) and age at strain isolation (y-axis).

Three quantities characterize the timing of events among strains and participants: i) ‘age-at-isolation’, which is the time separating strain isolation and participant birth (i.e., (year of strain isolation) – (year of participant birth)). This quantity can be negative if isolation occurred prior to Birth (B group). Corresponding measurements then reflect cross-reactivity as individuals cannot have directly encountered these strains (Figure 1a); ii) ‘age-at-sampling’, which is the time period separating participant birth and sampling (i.e., (year of sampling conducted) – (year of participant birth), always positive); and iii) ‘time-since-isolation’, which is the time interval between sampling and strain isolation (i.e., (year of sampling conducted) – (year of strain isolation)). Measurements mapping to positive ages at isolation and negative times since isolation represent responses to viruses circulating in the future (Future viruses, i.e., viruses isolated after sampling (F group)), after participant samples were taken and thus must also reflect cross-reactivity (see Figure 1a). The remaining measurements were for strains that participants may have encountered during their lifetime and were isolated before samples were taken (isolation during Lifetime and before sampling (L group)) such that antibody titers must reflect a mixture of cross reactivity and strain specific responses (see Figure 1a). Note that the same participant can be allocated to different groups for different strains, depending on the date of the strain isolation, the date of individual birth and date of sampling (see Figure 1b).

### Models of age, sex, strain and HI titers

To assess sex differences, we built upon published models of HI titer in this cohort [17] and fit a generalized additive model (GAM) to explain observed HI titers. Undetectable HI responses at the lowest dilution were assigned titers of 5, and all titers are transformed to the log_2_-scaleusing the formula y=log_2_(x/5) where x is the measured titer, resulting in a 0 for undetectable titers and a 1 unit rise in titer for each 2-fold increase (so 10=1, 20=2, 40=3, etc) [21].

A mixed effect GAM model with a zero inflated Poisson link estimated how HI titers depend on the interplay between age at sampling, age at isolation and sex. The model also includes a strain-specific intercept and a sex-specific intercept. This model captures the hypothesis that the interaction effect between age and age at isolation on measured HI titers is sex specific

Equation (1) $$log\;y_{ij}=\alpha_{j}+\beta_{i}+\gamma_{g_{i}}+S_{g_{i}}\left(p_{i},p_{ij}\right),$$

where *y*_*ij*_ is the observed HI titer of person *i* against strain *j*, *g*_*i*_ is the sex of the *i*-th person, *α*_*j*_ is the strain-specific intercept, *β*_*i*_ is the random effect of individual person *i* as coming from a Gaussian distribution with a mean of zero and an estimated variance, *γ*_*gi*_ is the sex-specific intercept, *p*_*i*_ is the age of the *i*-th person at sampling, *q*_*ij*_ is the age of the *i*-th person when the *j*-th strain circulated and $S_{g_{i}}\left(a,b\right)$ is the value of the interaction term between *a* and *b* of a basis smoothing spline with sex-specific (*g*_*i*_) shape.

### Model fitting and evaluation

Models were fitted and evaluated using the “gam” function in the mgcv (cran.r-project.org/web/packages/mgcv/), and the marginal effects package (https://cran.r-project.org/web/packages/marginaleffects/index.html).

## Results

### Summary metrics of antibody titers

**Figure 2.**
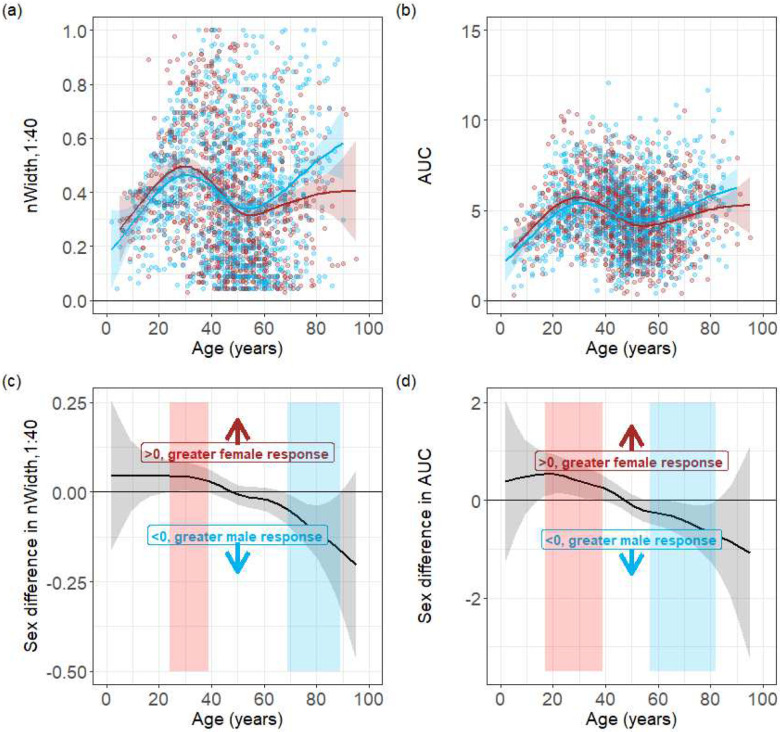
Summary metrics of antibody profiles by age and sex. Figures show the width above a titer of 1:40 (a), and the area under the curve (AUC) (c) by sex. Metrics developed by Yang et al. 2021 [18] were calculated using all strains (i.e., pre-birth strains and post-birth strains and normalized by the total number of strains tested by the individual. Blue and red represent the metrics measured for serum collected from males and females, respectively. Solid lines are predictions of sex differences from a generalized additive model, and the grey band represents the corresponding 95% confidence band. (b,d) show the model-predicted difference between sexes of width for titers ≥1:40, and the area under the curve (AUC) between sex, respectively. Lines with associated 95% confidence intervals above zero indicate a female bias while below zero represent a male bias. Significant differences e.g., the confidence intervals do not include 0, are marked with red areas when females have higher responses and blue areas when males have higher responses.

We found that females aged 25 to 39 years have statistically significantly wider widths with protective titers (≥1:40) (Figure 2a–b) and detectable titers (≥1:10) (aged 16 to 30 years, Figure S2a-b) than males of the same age ranges. We also found that females aged 15 to 39 years had statistically significantly higher ‘AUC’ in HI responses (Figure 2c–d) compared to males.

In contrast, we found that older males aged 69 to 89 years have statistically significantly wider widths of protective titers than females of the same age. Males aged 55 to 70 years had larger widths for detectable titers than females of the same age. Males aged 57 to 83 years of age also had statistically significantly higher AUC than females of the same age. This suggests that sex, interacting with age, modulates the totality of HI responses to a spectrum of influenza strains.

### The effects of sex, age and strain on HI titers

**Figure 3.**
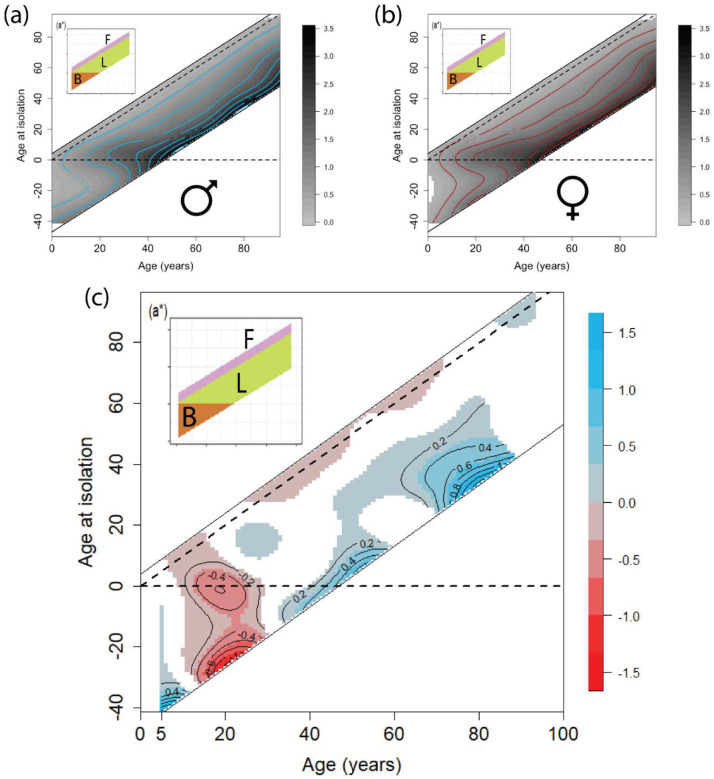
Model-predicted HI titers on a log_2_ scale for males (a), females (b) and sex differences (c) by age (x axis) and age at isolation (y axis). Contour intensities in Figure 3 (a, male) and Figure 3 (b, female) indicate titer magnitudes on a log_2_ scale while contour intensities in Figure 3 (c) indicate differences in male and female titers, where significant. The horizontal dashed black line shows age zero at isolation while the diagonal dashed black line shows the time of sampling. Figure 3 (a*) depicts the display of the three groups shown in Figure 1 (b). The predictions were constrained to the age range of 5 to 96 due to low sample size outside of this age range (see Figure S1). Although individual random effects are fitted in this model, the plots depict the average population-level effect. In Figure 3 (c), red depicts higher titers in females while blue depicts higher titers in males.

Based on previous work, an individual’s age is strongly associated with antibody titers against influenza A/H3N2 strains [17]; but it is not known whether there are sex differences in these relationships. Predicted HI titers on a log_2_ scale given individual age at sampling and age at isolation show broad consistency in males (Figure 3a) and females (Figure 3b): titers were high for viral strains for which participants had an age at isolation around zero (see the boundaries between B group and L group) and for the strains circulating during people’s lifetime and before samples were taken (L group). As is observed in other studies, HI titers are also elevated in older individuals. The detectable titers in both sexes of B group and F group indicate an unequivocal effect of cross-reactivity while augmented titers in L group are accounted for by the mixture effect of strain-specific responses and cross-reactivity. Both the cross-reactivity and strain-specific responses speak to the antigenic similarity between strains isolated within a few years of each other, i.e., lower titers are obtained in both males and females to strains distant from those experienced over their lives (e.g., strains circulating long before they were born); and higher titers were obtained to strains experienced in later life. In both sexes, forward cross-reactivity (i.e., titers to future strains that circulate after sampling conducted, F group) is weaker than backward cross-reactivity (B group of measurements).

### Sex difference of model-predicted log HI titers

To explore sex differences by age in these models, we plotted the difference in titer between sexes estimated by the model (Figure 3c), using the R package “*marginaleffects*”. Only combinations where the model-predicted 95% confidence interval of titer differential between the sexes is significantly different from zero are indicated, and darker colored areas indicate greater titers in red for females, blue for males (Figure 3c). Females generally have higher cross-reactive antibody responses than males. Females aged between ~10 and 32 in the B group, and aged 10–20 and 22–73 in the F group exceed male titres, with up to 40% higher HI titres in females aged 30–49 when averaged across both F strains (see Figure S3). However, males (particularly those over 50 years of age) had higher titers against those viruses that participants could have been exposed to over their lifetime (prior to sampling; (L group) compared to females of the same ages (Figure 3c, noting that older participants (e.g., >85 years old) are sparse in this study, see Figure S1). Males aged around 56–57 generated 10% higher antibody titers than females of the same age when averaging across all strains. The marginal effects of age at sampling and age at isolation are presented in Figures S4–6.

## Discussion

We identified substantial sex differences and their associations with age in the humoral immune response to strains of influenza A/H3N2, with females showing consistently stronger cross-reactivity between ages of post-puberty and perimenopause (approximately between 10 and 50 years old; B group). Older males (aged 50+), on the other hand, had stronger antibody responses against strains that they had potentially been infected by during their life course.

Although cross reactivity against historical influenza strains gained via natural infections has been studied in ferrets [22] and humans [23], we know of no previous work identifying the divergent age-associated patterns between the sexes identified here. Nevertheless, the patterns we identify aligns with known variations of immunological differences between the sexes throughout the course of life, especially those modulated by cyclical changes in sex steroids. For example, although infant males may produce higher inflammatory responses than females, after puberty inflammatory responses are consistently higher in females than in males. Females continue to have higher CD4^+^ T cell counts and higher CD4/CD8 T cell ratios than males throughout adulthood [2,19]. Affinity maturation of females has been found to yield lower specificity [24], which would align with expectations for greater cross-reactivity.

Our analysis has a number of limitations. Although we focused our analysis on interpreting potential biological differences between sexes in immunological response to influenza, epidemiological factors that may give rise to sex differences in exposure to influenza may contribute to our results [25]. Further, the interplay of these two phenomena (i.e. increased exposure leads to higher prevalence of antibody in one sex, leading to reduced infection in future time points and thus reduced antibodies to future strains), leads to difficulties in disentangling the role of differences in exposure and differences in immunological response. While the results provide insight into influenza immunology, our measurements are of antibody titers and thus B-cell driven responses; however, it is known that T-cell immunity also differs by sex in the context of influenza (e.g., males also had higher frequencies of flu-specific lung TRM compared with females [26]), and this might be an important source of vulnerability to infection and responses to antigen exposure, via vaccination or otherwise. Finally, our study measures only one aspect of the antibody response, the binding of influenza hemagglutinin. The coupling of responses across multiple pathways of immune responses to influenza may lead us to find differences in this one facet of the immune response by sex without recognizing that other responses offset these differences.

This cross-sectional study, including a wide age range of individuals tested against a large collection of strains circulating from 1968 to 2014, allows us to discriminate between antibody responses to strains that individuals had never experienced compared to strains that individuals could have been infected by at various ages. This opens the way to pinpointing effects solely due to cross-reactivity by separation of this data into components reflecting on the one hand, the measurement of antibody titers to strains that the individuals have lived through, and on the other hand, the cross-reactivity to strains that they have not emerge in the future, as remarked recently in a cohort study of young children [13]. Our study both deepens our fundamental understanding of sex differences in immune function and yields insights for vaccine formulation and administration. The known correlation between HI titers (especially above 1:40 antibody threshold) and protection against infection [27], indicates the possibility of an age-related female advantage; but expanding this requires more evidence on how antibody titers shape patterns of mortality beyond influenza [28–29].

The consistent sex differences we find may have relevance to the design of interventions including vaccines. Further work is needed to identify mechanisms underpinning these observations. However, if mechanisms of sex differences in response to influenza could be identified, they might be leveraged to deliver sex and/or age-specific vaccines of improved efficacy over generally targeted products.

## Supplementary Material

This is a list of supplementary files associated with this preprint. Click to download.

- SIflusex.pdf
